# miR-125a, miR-139 and miR-324 contribute to Urocortin protection against myocardial ischemia-reperfusion injury

**DOI:** 10.1038/s41598-017-09198-x

**Published:** 2017-08-21

**Authors:** Ignacio Díaz, Eva Calderón-Sánchez, Raquel Del Toro, Javier Ávila-Médina, Eva Sánchez de Rojas-de Pedro, Alejandro Domínguez-Rodríguez, Juan Antonio Rosado, Abdelkrim Hmadcha, Antonio Ordóñez, Tarik Smani

**Affiliations:** 1 Grupo de Fisiopatología Cardiovascular, Instituto de Biomedicina de Sevilla-IBiS, Universidad de Sevilla/HUVR/Junta de Andalucía/CSIC, Seville, 41013 CIBERCV Spain; 2 Departamento de Fisiología Médica y Biofísica, Universidad de Sevilla, Seville, 41009 Spain; 3 Departamento de Fisiología, Universidad de Extremadura, Caceres, 10071 Spain; 4 Center for Molecular Biology and Regenerative Medicine-CABIMER, Unversity of Pablo de Olavide-University of Seville-CSIC, Seville, Spain., Seville, 41010 CIBERDEM Spain

## Abstract

Urocortin 1 and 2 (Ucn-1 and Ucn-2) have established protective actions against myocardial ischemia-reperfusion (I/R) injuries. However, little is known about their role in posttranscriptional regulation in the process of cardioprotection. Herein, we investigated whether microRNAs play a role in urocortin-induced cardioprotection. Administration of Ucn-1 and Ucn-2 at the beginning of reperfusion significantly restored cardiac function, as evidenced *ex vivo* in Langendorff-perfused rat hearts and *in vivo* in rat subjected to I/R. Experiments using microarray and qRT-PCR determined that the addition of Ucn-1 at reperfusion modulated the expression of several miRNAs with unknown role in cardiac protection. Ucn-1 enhanced the expression of miR-125a-3p, miR-324-3p; meanwhile it decreased miR-139-3p. Similarly, intravenous infusion of Ucn-2 in rat model of I/R mimicked the effect of Ucn-1 on miR-324-3p and miR-139-3p. The effect of Ucn-1 involves the activation of corticotropin-releasing factor receptor-2, Epac2 and ERK1/2. Moreover, the overexpression of miR-125a-3p, miR-324-3p and miR-139-3p promoted dysregulation of genes expression involved in cell death and apoptosis (BRCA1, BIM, STAT2), in cAMP and Ca^2+^ signaling (PDE4a, CASQ1), in cell stress (NFAT5, XBP1, MAP3K12) and in metabolism (CPT2, FoxO1, MTRF1, TAZ). Altogether, these data unveil a novel role of urocortin in myocardial protection, involving posttranscriptional regulation with miRNAs.

## Introduction

Ischemia induced by the interruption of heart blood flow evokes significant cardiac myocytes damages. Paradoxically, the subsequent reperfusion also activates various injury responses and further tissue lesions what is known as ischemia and reperfusion (I/R) injuries^1^. Advances in the understanding of pathophysiological mechanisms of I/R injury has identified a number of possible therapeutic targets and cardioprotectors such as ligands that activate *RISK* (reperfusion injury salvage kinase) or *SAFE* (survival activating factor enhancement) pathways; metoprolol, beta1-selective blocker; or cyclosporine-A among others drugs as reviewed recently^2^. Urocortin isoforms (Ucn-1, Ucn-2, and Ucn-3) have emerged as potential therapeutic agonists that improve heart performance and protect it from I/R injuries^3, 4^. In the cardiovascular system, urocortin binds to a G-protein-coupled receptor called CRF-R2 (corticotropin releasing factor receptor-2), whose activation enhances cAMP intracellular concentration^5^, classically related to PKA activation. Now, it’s known that cAMP also activates Epac (exchange protein activated by cAMP), important regulator of several cAMP-regulated processes in heart^6^. We have previously shown that the addition of Ucn-1 improved intracellular Ca^2+^ concentration ([Ca^2+^]_i_) handling during I/R[7], and efficiently protected hearts from I/R damages by the modulation of apoptotic genes, CD40lg, Xiap and BAD, through the activation of Epac2 and ERK1/2^8^.

Recently, microRNAs (miRNAs) have emerged as important regulators of cardiovascular physiopathological processes^9^. miRNAs are non-coding single-stranded small RNA molecules with typically 18–25 nucleotides, acting as negative regulators of gene expression by inhibiting the translation of mRNA and/or promoting mRNA degradation by complementary base-pairing with 3-untranslated regions (3-UTRs). miRNAs are involved in regulating cardiac function, such as contraction, heart growth and morphogenesis. Interestingly, several miRNAs modulate the expression of hundreds of key proteins involved in I/R injury and myocardial infarct^10^. For example, miR-21 is upregulated by ischemic postconditioning in mouse hearts, where it played a protective role through PTEN/Akt signaling pathway^11^. miR-133a and miR133b are implicated in cardioprotection during ischemic postconditioning^12^, and remote ischemic preconditioning^13^. In addition, miR-125b protected heart from I/R injury by the prevention of apoptotic signaling^14^, meanwhile miR-499 mediates cardiac protection against I/R injury by targeting the called programmed cell death 4 (PDCD4) during ischemia postconditioning^15^.

In the present study, we explored the involvement of miRNAs in the regulation of urocortin-induced heart protection. We observed that Ucn-1 differentially regulates the expression of miR-125a-3p, miR-324-3p and miR-139-3p whose overexpression modulates several genes associated with a wide range of heart functions as cell stress, metabolism, cell survival and apoptosis.

## Results

### Urocortin-1 applied at the onset of reperfusion recovers hemodynamic performances in perfused rats hearts

**Figure 1:**
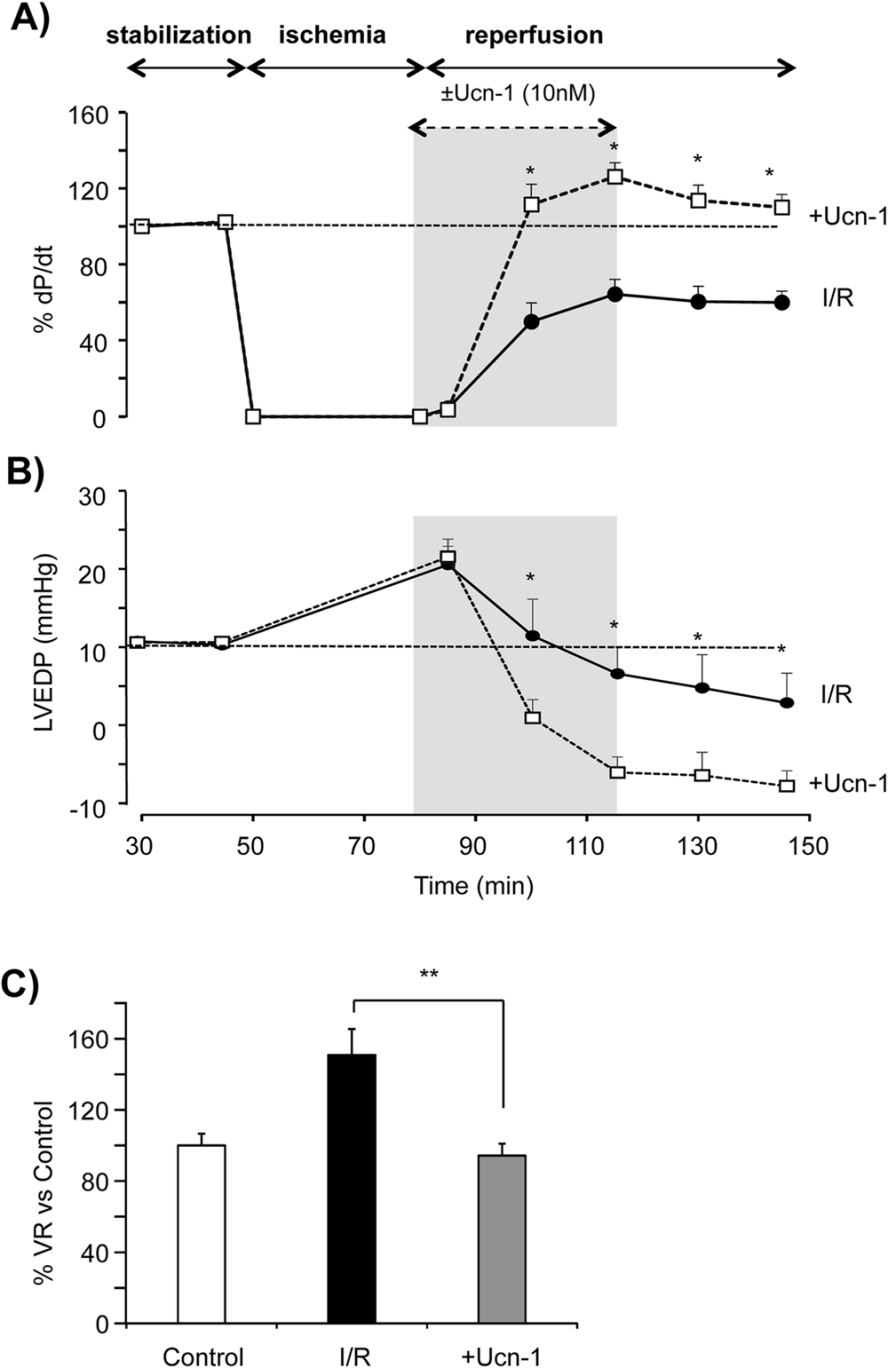
Urocortin-1 recovers the hemodynamic function of Langendorff perfused rat hearts submitted to I/R. (**A**) Graph shows summary data of the contractility expressed as +dP/dt (%) in hearts subjected to ischemia/reperfusion protocol (I/R, full circle, n = 5), and in hearts treated with 10 nM Ucn-1 (+Ucn-1, open square, n = 5). Ucn-1 was applied 5 minutes before ischemia ends and during the first 30 minutes in reperfusion as indicated by area in grey. (**B**) Shows summary data of the left ventricular end diastolic pressure (LVEDP, mmHg) in the same conditions as in “A”. (**C**) Bar graph shows the changes in the mean of vascular resistances (VR, mmHg*min/ml). Values were taken 60 minute after reperfusion and were normalized to controls’ values. Data are means ± S.E.M. “*” and “**” indicate significance at p < 0.05 and p < 0.01 of I/R vs +Ucn-1.

The protective effect of Ucn-1 on heart contractility was examined *ex vivo* in Langendorff-perfused rat hearts submitted to I/R. First, Table 1 shows that, in control hearts, the addition of 10 nM Ucn-1 increased significantly heart contractility examined by the maximum derivative of left ventricular pressure (dP/dt) and the left ventricular pressure (LVP), meanwhile it decreased the left ventricular end diastolic pressure (LVEDP) and the coronary vascular resistances (VR). Next, Fig. 1A shows that hearts exposed to 40 minutes of ischemia and 60 minutes of reperfusion (I/R) showed a significant decrease in dP/dt, indicating prominent loss in heart contractility. However, the administration of 10 nM Ucn-1, 5 minutes before reperfusion and maintained during 30 minutes, recovered completely heart’s contractility. The analysis of others hemodynamic parameters indicates that Ucn-1 improved the LVEDP (Fig. 1B), and the VR (Fig. 1C), whose progressive rise observed in reperfusion was prevented significantly by Ucn-1. Interestingly, the effects of Ucn-1 on heart performances were even maintained 60 minutes after washing out the peptide. Altogether, these data confirm that Ucn-1 addition at the onset of reperfusion recovers heart contractility, increases the ventricular diastolic loading and prevents coronary artery vasoconstriction, preserving effectively hearts’ performances.

**Table 1:** Summary data of changes in hemodynamics parameters produced by 10 nM Ucn-1 in Langendorff-perfused hearts.

Hemodynamics Paramaeters	Control	Ucn-1 (10 nM)
LVP (mmHg)	75.4 ± 5.6	130.9 ± 3.3
LVEDP (mmHg)	10.4 ± 0.1	−4.6 ± 2.9
+dP/dt (%)	100	195.9 ± 16.6
VR (%)	100	78.0 ± 6.5

Left ventricular pressure (LVP), left ventricular end diastolic pressure (LVEDP), maximum derivative of left ventricular pressure (+dP/dt), and vascular resistance (VR) were evaluated 20 minutes after Ucn-1 addition in 6 hearts.

### Urocortin-1 regulates differentially the expression of cardiac miRNA in perfused hearts

**Figure 2:**
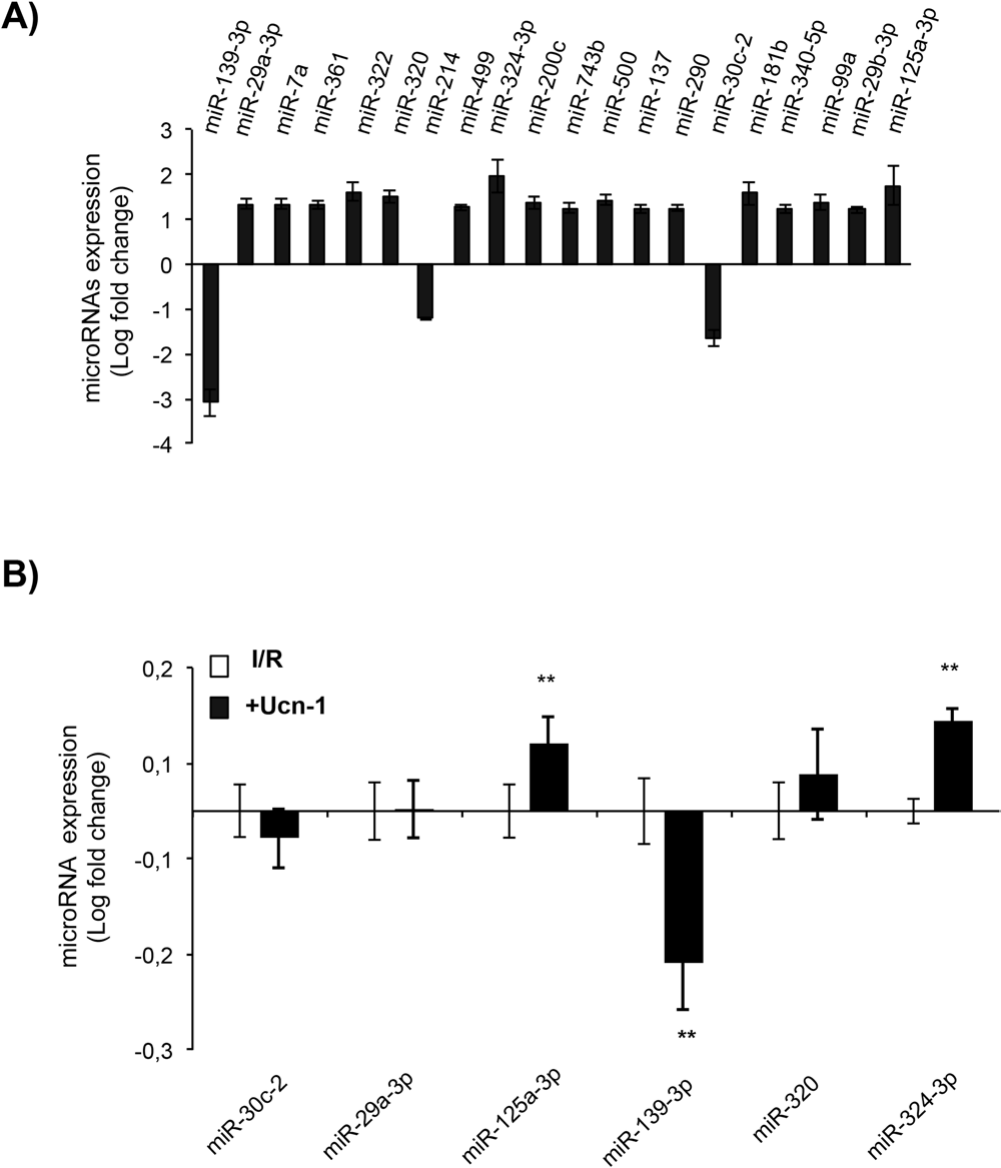
miRNAs detected by gene chip and qRT-PCR in perfused rat hearts. (**A**) List of up- and downregulated miRNAs detected by microarray in samples from Langendorff-perfused heart treated with Ucn-1 (10 nM) versus I/R (n = 4). (**B**) qRT-PCR conducted with samples from Langendorff-perfused heart treated with Ucn-1 vs I/R showing significant changes in the expression of miR-125a-3p, miR-139-3p and miR-324-3p but not of miR-30c-2, miR-29a-3p, or miR-320. Values are shown in logarithmic scale and are means ± S.E.M, (n = 6). “**” indicates significance at p < 0.01 as compared with I/R.

Once the cardioprotective effect of Ucn-1 was confirmed, we sought to determine whether Ucn-1 (10 nM) might regulate post-transcriptional processes involved in cardioprotection during I/R. We performed a miRNA array analysis in hearts’ samples taken 1 hour after reperfusion from Langendorff-perfused hearts undergoing the same protocol as in Fig. 1. Table 2 shows that Ucn-1 promoted differential expression of several miRNAs. 29 of them were up and 9 were downregulated based on the threshold of fold change >1.3. The expression of 20 of these miRNAs was significantly different at p < 0.05 as shown in Fig. 2A.

**Table 2:** miRNAs identified to be dysregulated.

Gene ID	Fold Change	*P* value
**miR-139-3p**	**−3.09**	**0.003**
miR-10a-5p	−2.28	0.110
**miR-324-3p**	**1.96**	**0.016**
miR-145	1.88	0.144
**miR-125a-3p**	**1.75**	**0.050**
miR-494	1.66	0.256
**miR-30c-2**	**1.64**	**0.035**
**miR-322**	**1.62**	**0.013**
**miR-181b**	**1.58**	**0.036**
**miR-320**	**1.51**	**0.014**
miR-221	1.47	0.085
miR-126	−1.47	0.224
**miR-500**	**1.44**	**0.027**
miR-140	1.42	0.146
miR-15b	−1.41	0.092
miR-99b	1.40	0.127
miR-466b	1.40	0.167
**miR-99a**	**1.39**	**0.042**
miR-128	1.38	0.226
**miR-200c**	**1.36**	**0.023**
miR-872	1.36	0.138
miR-450a	1.36	0.092
miR-24-2	1.35	0.076
miR-30e	1.35	0.053
miR-196c	1.35	0.067
miR-152	-1.35	0.253
**miR-7a**	**1.34**	**0.010**
**miR-29a-3p**	**1.34**	**0.010**
miR-324-5p	1.33	0.097
miR-598-3p	1.33	0.079
**miR-361**	**1.32**	**0.011**
**miR-214**	**−1.31**	**0.015**
let-7d	1.31	0.338
miR-25	1.31	0.093
miR-199a-5p	−1.30	0.378
miR-342-3p	−1.30	0.097
**miR-137**	**−1.30**	**0.034**
**miR-340-5p**	**1.30**	**0.030**

List of expressed miRNAs defined as dysregulated in miRNA microarray analysis in the Ucn-1 group as compared with the I/R group. miRNAs are classified by higher rate of change in Ucn-1 treatment versus untreated I/R. Expressed miRNAs are filtered based on “Fold change >1.3 or <−1.3”. Bold letters is for miRNAs with significant changes at p < 0.05. I/R, ischemia/reperfusion; Ucn-1 (10 nM) was applied 5 minutes before reperfusion.

To validate microarray results, we further examined the expression of miRNAs using qRT-PCR in samples from Langendorff-perfused hearts undergoing the same protocol as in Fig. 1. We focused on 6 miRNAs, miR-30c-2, miR-29a-3p, miR-125a-3p, miR-139-3p, miR-320, and miR-324-3p, which role in cardioprotection is still unknown. As shown in Fig. 2B, Ucn-1 upregulated significantly the expression of miR-125a-3p and miR-324-3p, meanwhile it downregulated the expression of miR-139-3p. Nevertheless, changes in the levels of miR-29a-3p, miR-30c-2, and miR-320 were not significantly affected by Ucn-1.

### Urocortin-1 modulates the expression of miR-125a-3p, miR-324-3p, and miR-139-3p in adult cardiac myocytes

**Figure 3:**
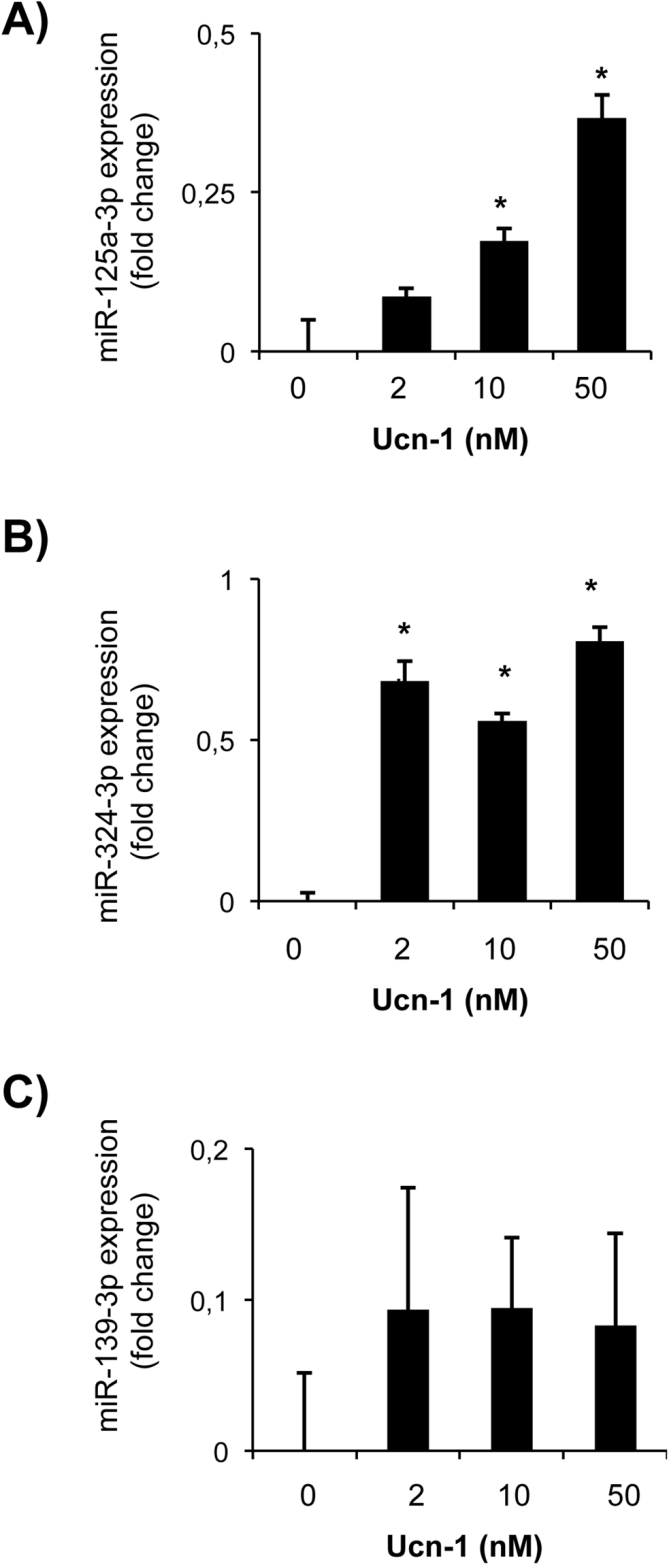
Urocortin-1 regulates miR-125a-3p, miR-324-3p and miR-139-3p expression in isolated cardiac myocytes. Bar graphs showing fold changes in the expression of miR-125a-3p (**A**), miR-324-3p (**B**) and miR-139-3p (**C**) in isolated cardiac myocytes treated 10 minutes with increasing concentrations of Ucn-1 (2, 10 and 50 nM). Values are means ± S.E.M, (n = 4). “*” indicates significance at p < 0.05.

**Figure 4:**
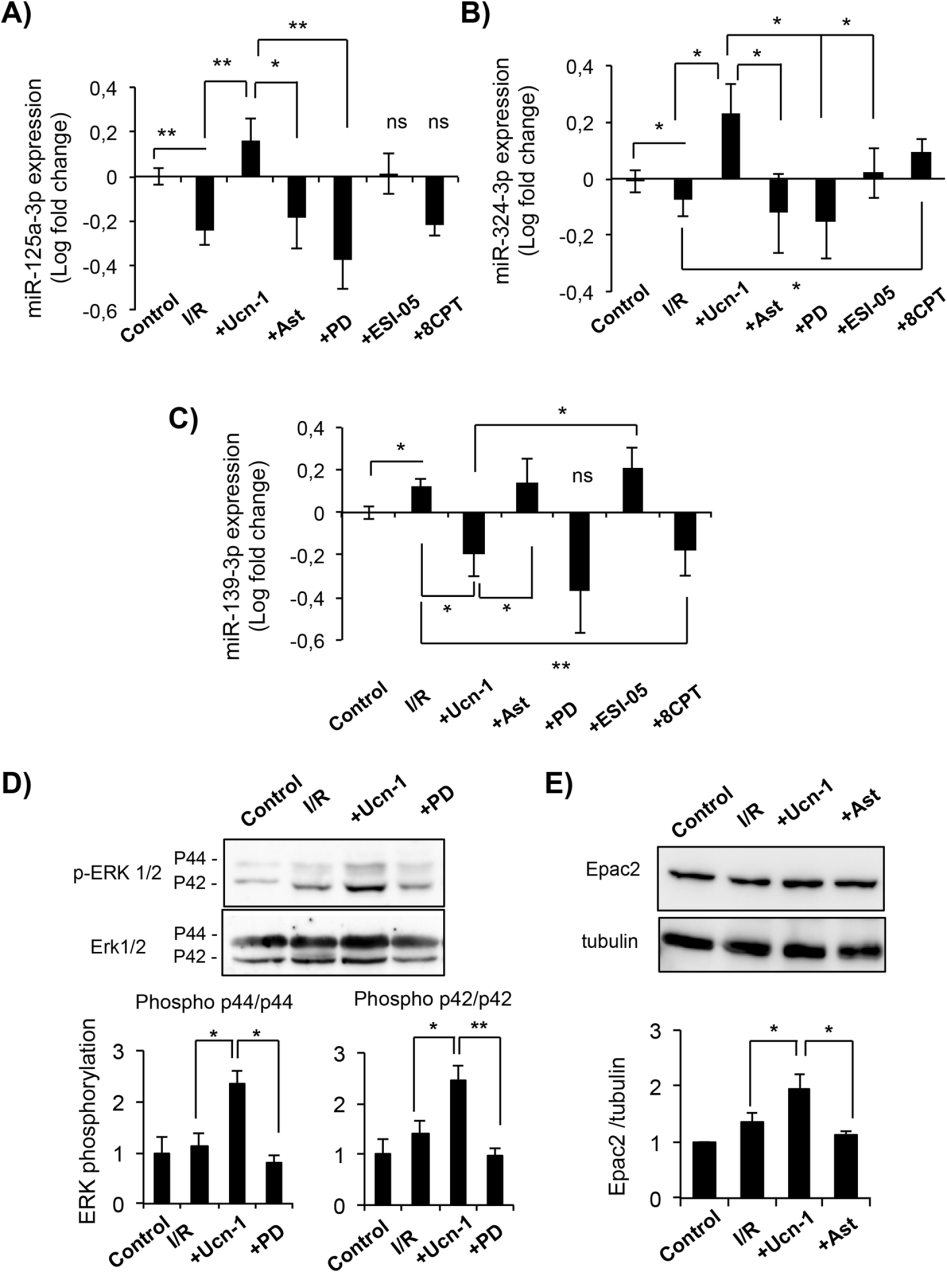
Urocortin-1 regulates the expression of miRNAs in isolated cardiac myocytes through ERK1/2 and Epac2 activation. (**A**–**C**) Bar graphs show changes in the expression of miR-125a-3p (**A**), miR-324-3p (**B**) and miR-139-3p (C) in isolated cardiac myocytes treated with Ucn-1 (10 nM) in reperfusion. (**D**) Western blot and summary data showing ERK 1/2 phosphorylation in cells exposed to I/R (30 minutes/10 minutes). (**E**) Western blot and summary data showing Epac2 examined in cells exposed to I/R (30 minutes/18 hours each). “I/R” is for cells undergoing I/R protocols. “+Ast” is for cells subjected to I/R pre-treated 10 minutes with 0.5 µM astressin to inhibit CRF-R2 before the addition of Ucn-1. “+PD” is for cells pre-treated 10 minutes with 5 µM PD 098059 to inhibit ERK1/2, before the addition of Ucn-1. “+ESI-05” is for cells pretreated 10 minutes with ESI-05 (10 µM) to inhibit Epac2, before the addition of Ucn-1. “+8CPT” is for cells treated with 10 µM 8CPT at the onset of reperfusion. Values are shown in logarithmic scale and are means ± S.E.M, (n = 6–9 independent cells preparation). “*” and “**” indicate significance at p < 0.05 and p < 0.01.

To assess the endogenous expression of miR-125a-3p, miR-324-3p, and miR-139-3p, we treated isolated cardiac myocytes with different concentrations of Ucn-1 (2, 10 and 50 nM). Figure 3 shows that the expression of miR-125a-3p and miR-324 are increased significantly upon treatment with Ucn-1. In contrast, the level of miR-139-3p did not change significantly in cardiac myocytes incubated with any of the used Ucn-1 doses. Next, we examined whether Ucn-1 may regulate the miRNAs expression in adult cardiac myocytes subjected to I/R protocol. We found that, after I/R, the expression of miR-125a-3p (Fig. 4A) and miR-324-3p (Fig. 4B) decreased, whereas the expression of miR-139-3p increased (Fig. 4C). Interestingly, cells treatment with Ucn-1 (10 nM) reverted the effect of I/R. Ucn-1 enhanced significantly the expression of both miR-125a-3p (Fig. 4A) and miR-324-3p (Fig. 4B), meanwhile Ucn-1 downregulated miR-139-3p (Fig. 4C), confirming the data obtained in perfused hearts (Fig. 2B). Furthermore, as shown in Fig. 4A–C, these regulations of miRNAs by Ucn-1 were prevented in cardiac myocytes pretreated with astressin (Ast, 0.5 µM), the specific antagonist of CRF-R2 receptor. Therefore, Ucn-1 regulated significantly the expression of miRNAs through a mechanism involving CRF-R2.

### Signaling pathway involved in urocortin-1 regulation of miRNAs

Recently, we have shown that ERK1/2 and Epac2 are involved in various cardioprotective effects of Ucn-1^8^. Therefore, we examined the implication of ERK1/2 and Epac2 signaling pathway in Ucn-1 regulation of miR-125a-3p, miR-324-3p and miR-139-3p. Figure 4D shows that Ucn-1 (10 nM) addition to isolated cardiac myocytes under I/R enhanced the phosphorylation of both ERK p42 and p44 isoforms, which was potently reduced by PD 098059 (5 µM), specific inhibitor of ERK1/2^8^. Interestingly, cells pretreatment with PD 098059 prevented Ucn-1 upregulation of miR-125a-3p (Fig. 4A) and miR-324-3p (Fig. 4B), but it did not affect miR-139-3p (Fig. 4C) expression levels during cells reperfusion.

We further investigated Ucn-1 effect on Epac. Fig. 4E shows that Ucn-1 significantly increased the expression of Epac2 under I/R, which was prevented completely in cells pretreated with astressin. Furthermore, Epac2 specific inhibition with ESI-05 (10 μM)^16^, blocked the effect of Ucn-1 on miR-324-3p (Fig. 4B) and miR-139-3p (Fig. 4C) regulation, but not miR-125a-3p expression (Fig. 4A). In addition, we found that the incubation of cardiac myocytes with 8CPT (10 μM), specific agonist of Epac1 and 2^16^, mimicked mostly the effect of Ucn-1. The administration of 8CPT prevented I/R-induced changes on the expression of miR-324-3p and miR-139-3p; meanwhile it did not affect the change induced by I/R of miR-125a-3p expression.

Altogether, these data suggest that Ucn-1 applied on the onset of reperfusion regulates differentially miR-125a-3p, miR-324-3p and miR-139-3p through ERK1/2 and/or Epac2 signaling pathways.

### Urocortin-2 infusion protects heart from I/R injuries and regulates the expression of miRNAs

**Figure 5:**
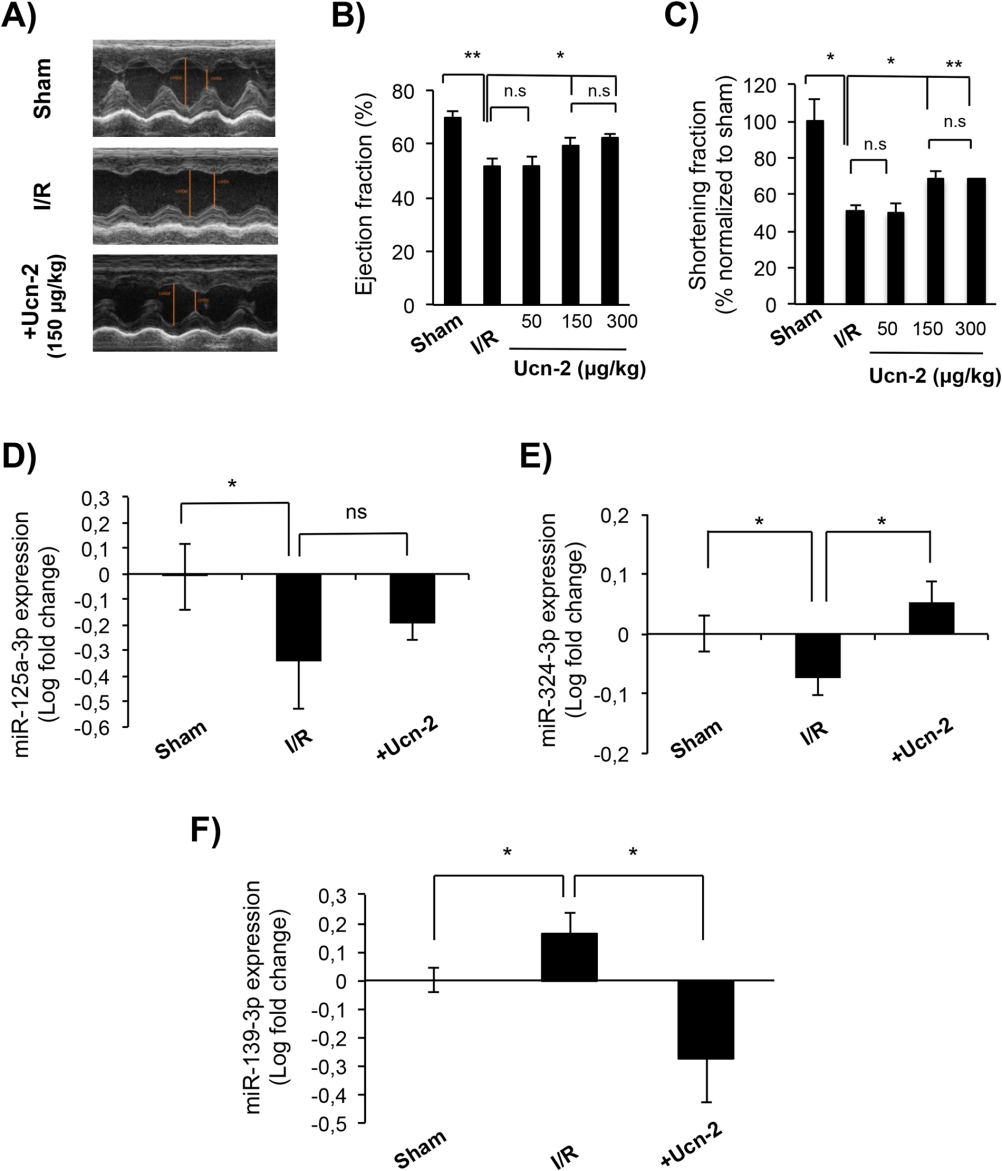
Infusion of urocortin-2 improves cardiac function in I/R and regulates the expression of miRNAs. (**A**) Cardiac function analyzed by echocardiography in M-mode 24 hours after surgery in three experimental models. Images are from “*Sham”* (n = 5); from “*I/R*” group corresponding to rats undergoing occlusion of the left coronary artery (LCA) for 40 minutes followed by reperfusion (n = 6); and for I/R rats infused with Ucn-2 (150 µg/Kg) 5 minutes before reperfusion (n = 5). (**B**,**C**) Bar graphs summarize ejection fraction (EF, %) and shortening fraction (SF, %) measured in rats infused with 50, 150 or 300 µg/Kg of Ucn-2. (**D**–**F**) Bar graphs showing the expression of miR-125a-3p, miR-324-3p and miR-139-3p, respectively in hearts from *Sham*, *I/R* animal model, and from I/R rats infused with Ucn-2 (150 µg/Kg; +Ucn-2). Values are shown in logarithmic scale. Data show means ± S.E.M. (n = 5–7). “*” and “**” indicate significance at p < 0.05 and p < 0.01, respectively.

To give more insight into the regulation of miRNAs by urocortin, we investigated its effect *in vivo* in rat experimental model of I/R provoked by transient LCA ligation. As Ucn-1 binds both to CRF-R1 and CRF-R2 expressed in the brain, in the heart and the peripheral organs; therefore, instead of Ucn-1, we infused Ucn-2 *i.v*. in tails’ rat 5 minutes before reperfusion to avoid its possible effects on central nervous system^4^. First, we confirmed that Ucn-2 and Ucn-1 have similar beneficial effects on contractility expressed as dP/dt and LVEDP in Langendorff-perfused heart (Supplement Fig. 1). Second, we evaluated the infusion of Ucn-2 at 50 µg/Kg, 150 µg/Kg and 300 µg/Kg on cardiac contractility in I/R rat model. Figure 5A shows that temporary ligation of LCA reduced significantly the contractile capacity of heart, as observed by M-Mode echocardiography 24 hours after surgery. As shown in Fig. 5B the ejection fraction (EF) in I/R group was significantly reduced (50.8% ± 3.2) as compared with *Sham* (70.1% ± 2.8); whereas, EF recovered significantly after the infusion of Ucn-2 at 150 µg/Kg (59.4% ± 3.1; Fig. 5A and B) and at 300 µg/Kg and (62.3% ± 1.2); but not when Ucn-2 was administrated at 50 µg/Kg (51.8% ± 3.5). Similarly, as illustrated in Fig. 5C the shortening fraction (SF) was reduced in I/R group (51.1% ± 8.1) comparing with *Sham*; whereas the infusion of Ucn-2 at 150 µg/Kg (68.1% ± 8.9) and at 300 µg/Kg (68.1% ± 1.1) recovered significantly the SF; but not when it was administrated at 50 µg/Kg (49.7% ± 5.4). Therefore, Ucn-2 was used at 150 µg/Kg to examine the expression of miR-125a-3p, miR-324-3p, and miR-139-3p in hearts’ samples taken 24 hours after surgery. Figure 5D and E shows that the expression of miR-125a-3p and miR-324-3p was downregulated in I/R group as compared to *Sham*. Ucn-2 infusion partially recovered the levels of miR-125a-3p (Fig. 5D); meanwhile, it reverted significantly the expression of miR-324-3p (Fig. 5E). In contrast, the expression of miR-139-3p was enhanced under I/R, but following the administration of Ucn-2 miR-139-3p was efficiently downregulated as illustrated in Fig. 5F. Altogether, these data confirm in I/R animal model that infusion of Ucn-2 right before reperfusion regulates the expression of, at least, significantly miR-324-3p and miR-139-3p.

### The transfection of cardiac myocytes with lentivirus overexpressing miRNAs alters the expression of several genes

**Figure 6:**
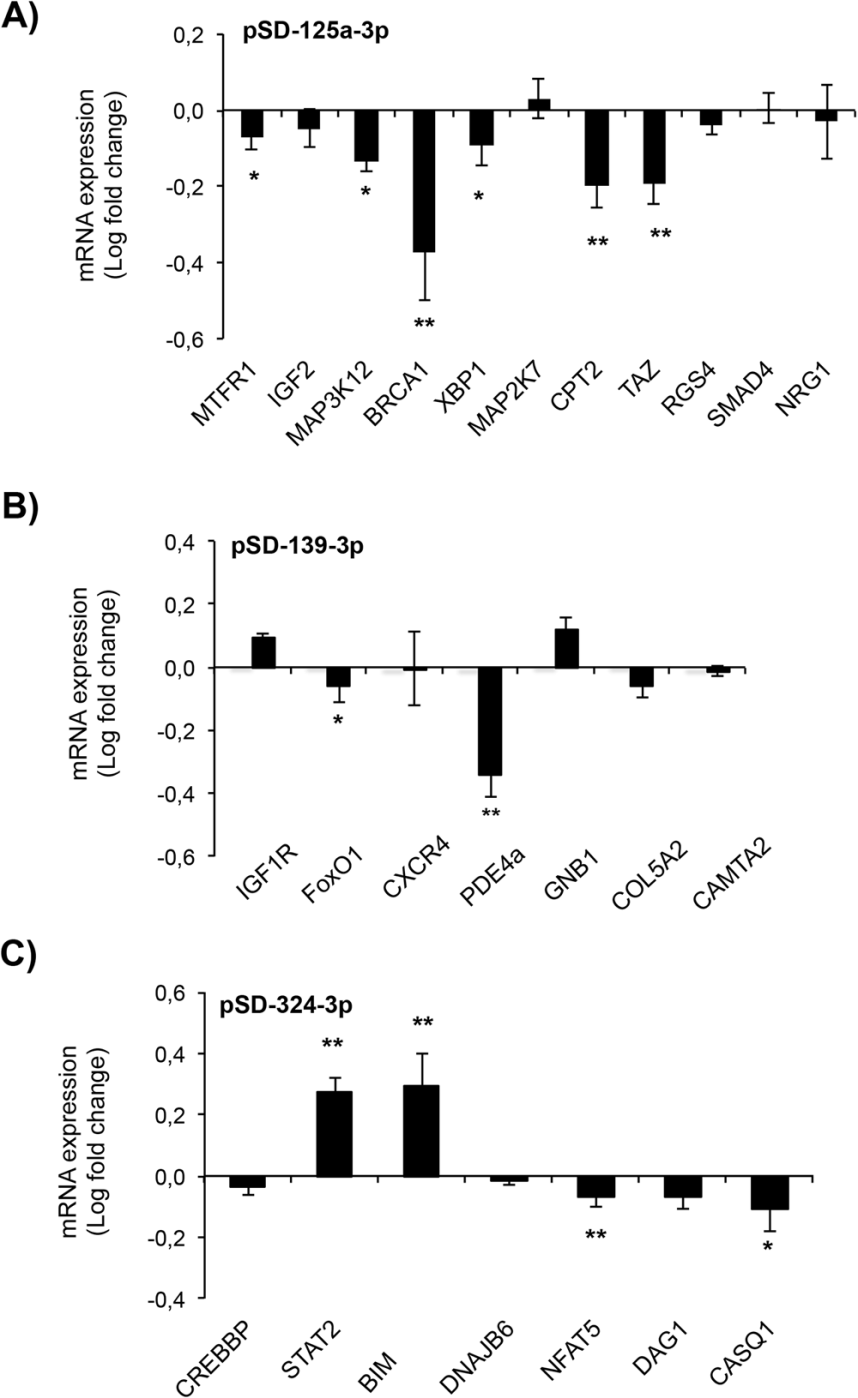
Quantification of mRNA expression by qRT-PCR of microRNAs targets genes. Bar graphs show rate changes of genes mRNA expression assessed by qRT-PCR in neonatal rat cardiomyocytes infected with lentivirus overexpressing miR-125a-3p (**A**), 139-3p-3p (**B**) and miR-324-3p (**C**). Values are shown in logarithmic scale compared to cells infected with the GFP vector. Values are means ± S.E.M (n = 8). “*” and “**” indicate significance at p < 0.05 and p < 0.01, respectively.

Using miRNA targets prediction, a group of candidate target genes were identified and selected for measurement of mRNA expression in neonatal rat ventricular cardiac myocytes (NRVMs) transfected with lentivirus overexpressing miRNAs. Table 1 shows a list of selected genes known to regulate different cardiac pathophysiological processes related to cardioprotection, apoptosis and cell survival (e.g. BRCA1, IGF1, MTFR1, NRG1, STAT2, …); to adverse remodeling and fibrosis (e.g. NFAT5, CAMTA2, CREBBP, MAP3K12, COL5A2, DNAJB6, …); or to cell metabolism and stress (XBP1, MTFR1, FoxO1, TAZ, …), among other actions. First, we examined whether Ucn-1, under I/R, changes the expression of miRNAs in NRVMs in the same way as in adult cardiac myocytes. Figure 2 confirms that the addition of Ucn-1 in reperfusion significantly upregulated the expression of miR-125-3p and miR-324-3p, meanwhile it downregulated miR-139-3p. Second, Fig. 6A shows that the transfection of NRVMs with lentivirus over-expressing miR-125a-3p (LV-miR-125a-3p) significantly downregulated the expression of BRCA1, CPT2, MAP3K12, MTFR1, TAZ, and XBP1. Similarly, Fig. 6B shows that cardiac myocyte transfection with LV-miR-139-3p significantly attenuated the expression of PDE4a and FoxO1. However, as shown in Fig. 6C, cell transfection with LV-miR-324-3p unusually upregulated the expression of STAT2 and BIM genes, meanwhile it downregulated NFAT5 and CASQ1.

**Figure 7:**
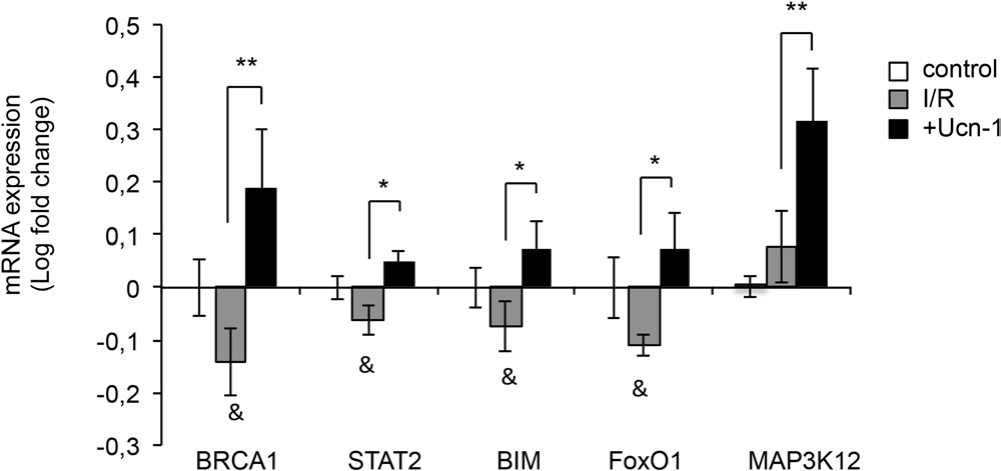
Urocortin-1 regulates the expression of BRCA1, STAT2, BIM, FoxO1 and MAP3K12 in isolated cardiac myocytes under I/R. Bar graphs show rate changes of genes’ mRNA expression assessed by qRT-PCR in adult rat cardiac myocytes (control); in untreated cells undergoing I/R protocols myocytes (*I/R*); and in cells treated with Ucn-1 in I/R (10 nM, +*Ucn1*). Values are in logarithmic scale normalized to control and are means ± S.E.M (n = 8). “*” and “**” indicate significance at p < 0.05 and p < 0.01, respectively. “&” indicates significance between I/R and control at p < 0.05.

Furthermore, we examined whether Ucn-1 regulates the expression of these genes in adult cardiac myocytes under I/R. Figure 7 shows that the expression of BRCA1, STAT2, BIM, FoxO1 and MAP3K12 decreased under I/R, meanwhile cells treatment with Ucn-1 in reperfusion significantly reversed the expression of these genes. Moreover, Ucn-1 evoked changes in others genes regulated by miR-125a-3p, miR-324-3p and miR-139-3p were not significantly different (data not shown). These data reveal that Ucn-1, through miR-125a-3p, miR-324-3p and miR-139-3p, might target multiple genes involved in different signaling pathway implicated in cardioprotection, which will induce synergetic beneficial effects.

## Discussion

Damages produced by I/R damage are known to occur mainly during the first minutes of reperfusion^17^. Therefore, concomitant myocardial I/R injuries still remains a major challenge even after a successful revascularization of an ischemic heart^1, 2^. I/R impairment of heart’s function is due to multifactorial and complex processes, including post-transcriptional gene regulation^9, 10^. Dysregulation of the expression of miRNA in the early phase of acute myocardial infarction (AMI) has been previously reported^18, 19^. Herein, we demonstrated that Ucn-1 and -2 addition at the onset of reperfusion efficiently protected hearts from I/R injuries and modulated the expression of several miRNAs. The efficient protection exerted by Ucn-1 was observed in different experimental models of I/R, including isolated adult cardiac myocytes and *ex vivo* perfused heart. Ucn-1 recovered completely heart contractility, reduced coronary vascular resistance, and decreased LVEDP, indicating that Ucn-1 prevents heart’s dysfunction when applied at the onset of reperfusion. These effects were consistent with Ucn-1’s action when it was applied both in preconditioning and postconditioning protocols^7, 8^. Furthermore, we have evaluated the effect of urocortin in animal model subjected to temporary LCA occlusion, using Ucn-2 instead of Ucn-1 to minimize the effect of Ucn-1 through CRF-R1 in the central nervous system^5^. Ucn-1 and Ucn-2 have recognized similar biological effects associated with stress, appetite and feeding behavior, and with vascular tone regulation or with muscle remodeling^20^. Here, we showed that one single *i.v*. administration of Ucn-2 in rats exposed to I/R improved significantly cardiac contractility, in agreement with other studies that showed the beneficial effect of the infusion of urocortin peptides in different set of strategies to improve heart failure^21–23^.

In the current study, we demonstrated that the addition of Ucn-1 or Ucn-2 in the beginning of reperfusion changed the expression of miR-125a-3p, miR-324-3p, and miR-139-3p in different experimental models of I/R. The changes of miRNAs expression were mild but still significantly different as compared with changes induced by reperfusion. Ucn-1 inverted the effect of I/R and enhanced the expression of miR-125a-3p and miR-324-3p, meanwhile it decreased miR-139-3p expression. Similarly, Ucn-2 infusion in the animal model of I/R modulated the expression of miR-324-3p and miR-139-3p, but it failed to recover completely miR-125a-3p levels. Interestingly, we found that Ucn-1 evoked miRNAs regulation through the activation of CRF-R2, ERK1/2, and Epac2, which is consistent with their role in cardioprotection^8, 24^. Here, we showed for the first time that Ucn-1 enhanced the expression of Epac2, and confirmed that it stimulated ERK1/2 under I/R. Remarkably, the upregulation of miR-125a-3p and miR-324 expression by Ucn-1 involved ERK1/2 activation; whereas, Epac2 was implicated in miR-139-3p and miR-324 regulation. ERK1/2 regulation of miRNAs has been previously addressed as it modulated the expression of miR-133a in cardiac myocytes^25^, and miR-145 in vascular smooth muscle cells^26^. In contrast, little is known about Epac2 regulation of miRNAs in the heart. Recent studies indicated that Epac modulated the expression of miRNAs in other cell types, as it is the case of miR-20 in macrophages^27^, and miR-451 in astrocytoma cells^28^.

The role of miR-125a-3p, miR-324-3p, and miR-139-3p in cardioprotection is still unclear. Previous reports demonstrated that circulating miR-125a-5p was associated with heart failure^29^, and it was significantly upregulated in early phase of reperfusion in mice^30^. An other recent study demonstrated that miR-139-5p is downregulated in left ventricular tissue isolated from patients undergoing remote conditioning^31^. Our data indicates that the upregulation of miR-125a-3p in cardiac myocytes changed significantly the expression of different genes whose functions are associated with cell stress, metabolism, or cell survival and apoptosis. We determined that miR-125a-3p inhibited the expression of BRCA1, a well-known tumor suppressor with multiple interacting partners and diverse biological functions^32^. In contrast, Ucn-1 increased BRCA1 expression under I/R in heart, which may be protective consistent with the role of BRCA1 in regulation of cardiac myocytes survival after myocardial infarction, its shielding of cardiac myocytes from DNA damage^33^, and its prevention of cardiac hypertrophy^34^. miR-125a-3p also reduced significantly the expression of MAP3K12 and XBP1, genes associated with cell stress. Meanwhile, Ucn-1 increased the expression of MAP3K12. MAP3K12, as a JNK signal pathway, is involved in apoptosis and is activated by exposure to environmental stresses such as oxidative stress^35^. XBP1 is a key regulator of sarcoplasmic reticulum stress found overexpressed in patients with ischemic cardiomyopathies^36^.

Furthermore, we observed that overexpression of miR-125a-3p inhibited others genes, as TAZ, CPT2 and MTFR1, related to mitochondria remodeling and metabolism. The inhibition of MTFR1 will be beneficial for heart protection, as it normally regulates mitochondrial fission and apoptosis in cardiac myocytes. Actually, MTFR1 knockdown reduced myocardial infarction sizes in mice^37^. A recent study suggested that impaired function of TAZ does not enhance susceptibility to ischemia-reperfusion injury^38^. Meanwhile, CPT2 downregulation disturbed the correct function of mitochondria metabolism in hearts, which will not help cells recovery in reperfusion^39^. In fact, decreased CPT2 activity was observed in ischemic heart, which promoted the production of reactive oxygen species in cardiac mitochondria^40^.

On the other hand, we found that miR-139-3p overexpression reduced significantly the transcription factor FoxO1, which is essential for sustaining cardiac cells metabolism and viability^41^. FoxO1 downregulation and its posttranslational modulation was also involved in the alteration of heart’s function, including collagen and protein metabolism dysregulation in a mouse model of post-ischemic heart failure^42^. Herein, we demonstrated that Ucn-1 decreased miR-139-3p’s levels and increased the expression of FoxO1 under I/R, which consequently will be beneficial for cardiac myocytes survival. In fact, FoxO1 reduced cardiac myocytes stress by upregulating antioxidant, anti-apoptotic, and autophagy genes^43^. miR-139-3p, but not Ucn-1, also inhibited the expression of PDE4a, a critical protein involved in cAMP hydrolysis and heart contraction^44^. PDE4 selective inhibitors were evaluated in the context of cardioprotection, but their effects were not conclusive^45^. They increased cAMP levels induced by catecholamine but they also promoted arrhythmias^46^. In this way, Ucn-1 induced miR-139-3p inhibition under I/R might be beneficial, as it will avoid the associated detrimental effects of PDE4 inhibition, although the effect of Ucn-1 on the expression of PDE4 needs further confirmation.

We also observed that miR-324-3p and Ucn-1 increased the expression of STAT2 and BIM (BCL2L11) genes; both of them involved in apoptosis and cardioprotection. Initially, miRNAs effect was thought to mediate downregulation in a one-way process, decreasing mRNA stability and/or translational inhibition. Nevertheless, as discussed recently, several evidences demonstrated that some miRNAs could upregulate genes expression in specific cell types and conditions with distinct transcripts and proteins^47^, consistent with our findings. Previous studies have demonstrated that different STAT members modulate the apoptotic program. STAT1 was involved in apoptosis and in pro-inflammatory process in cardiac myocyte under I/R^48^; and STAT3 was implicated in Ucn-1 mediated cardioprotection^49^. However, little is known regarding the role of STAT2 in cardioprotection. In the same way, the increase of BIM expression by miR-324-3p suggests the initiation of apoptosis by Ucn-1, which is reliable with our recent finding demonstrating that Ucn-1 enhanced the expression of a pro-apoptotic gene, BAD, under I/R^8^. In contrast, other reports have shown that BIM is downregulated by miR-24^50^, and miR-221^51^, which inhibited apoptosis. The beneficial role of apoptosis in detriment of necrosis to reduce the impact of I/R lesions has been largely discussed^52^. Apoptosis is a highly regulated energy-consuming process required for controlled and programmed cell death, which will certainly be beneficial to limit cardiac cell loss and posterior inflammation processes in I/R. Moreover, miR-324-3p overexpression downregulated NFAT5 and CASQ1 genes, whose role in cardioprotection are unknown. Although, NFAT5 expression was increased during protection against hypertonicity during I/R in rat kidney^53^; whereas, CASQ1 is a calcium-regulating protein involved in cardiac myocyte contraction, whose alteration was associated with Ca^2+^ overload during I/R^54^.

To conclude, this study highlights unidentified effects of Ucn-1 on transcriptional and posttranscriptional processes in heart subjected to I/R. Ucn-1 affords acute cardioprotective effects, but also modulates the expression of miR-125a-3p, miR-324-3p, and miR-139-3p, whose overexpression alters the expression of different genes. Further experiments will be important to corroborate the potential of these miRNAs as therapeutic targets for cardioprotection that might be a useful strategy for long-term heart protection.

## Materials and Methods

All the experiments with animals were performed in accordance with the recommendations of the Royal Decree 53/2013 in agreement to the Directive 2010/63/EU of the European Parliament and approved by the local Ethics Committee on human Research of the “Virgen del Rocio” University Hospital of Seville and the Animal Research Committee of the University of Seville.

### Global ischemia/reperfusion (I/R) in Langendorff-perfused rat heart

Adult male Wistar rats weighing 250–300 g were anaesthetized by intraperitoneal administration of an overdose of sodium thiopental (200 mg/Kg) as described previously^7^. The hearts were quickly removed, mounted on the aortic cannula of the Langendorff perfusion system apparatus and perfused with an oxygenated Krebs-Henseleit (KH) buffer (118 mM NaCl, 4.7 mM KCl, 1.25 mM CaCl_2_, 1.2 mM KH_2_PO_4_, 1.2 mM MgSO_4_, 25 mM NaHCO_3_, and 5 mM glucose) as described previously^7, 8^. The buffer was equilibrated with 95% O_2_ and 5% CO_2_. To obtain an isovolumetrically beating preparation, a latex balloon filled with water and connected by a catheter to a transducer (Abbott, IR), was inserted through the left atrium into the left ventricle and inflated to provide an end-diastolic pressure between 8 and 12 mmHg. Before starting with experimental protocols, the isolated hearts were set at a mean arterial pressure of 60–80 mmHg and were allowed to stabilize at 37 °C for 40 to 60 minutes. Chart Powerlab software (AD Instruments) was used for continuous recording throughout the experiments of heart rate, and maximum positive derivative of left ventricular pressure (+dP/dt). The heart contractility under different treatments was evaluated, in rate-paced hearts, by the analysis of +dP/dt, which corresponds to percent (%) increase of +dP/dt normalized to basal +dP/dt after stabilization period. I/R was conducted in isolated hearts by closing the perfusion to hearts and period of reperfusion started by the restoration of the flow.

To evaluate Ucn-1 or -2 effect on heart undergoing I/R, we considered the following experimental groups: Group 1 (ischemia/reperfusion, I/R): after stabilization, hearts were exposed to global ischemia during 40 minutes followed by 1 hour reperfusion with freshly oxygenated KH solution. Group 2 (I/R + Ucn-1 or Ucn-2): 10 nM of Ucn was applied at the beginning of reperfusion using an external pump with low flow. Urocortin was maintained during the first 30 minutes of reperfusion. Hearts were kept in reperfusion during additional 90 minutes.

### Rat model for myocardial ischemia and reperfusion (I/R)

Male Wistar rats weighing 250 ± 50 grams were anesthetized with a mixture of O_2_/sevoflurane 2%, then with anesthesia (50 mg/kg ketamine plus 8 mg/kg xylazine i.p) as described previously^54^. A small animal ventilator was used (Harvard Apparatus, Model 683, Massachusetts, NC) with a tidal volume (Vt) of 1.60–2 ml and 75–80 ventilations per minute. A left thoracotomy was performed in the intercostal space between the third and fourth ribs, followed by a pericardiotomy. The left coronary artery (LCA) was occluded with a 6-0 Prolene^TM^ silk suture (Ethicon, Spain) and tied off below the level of the left atria appendage and over a small tube placed into the suture for the posterior release of the occlusion, for a correct reperfusion. LCA occlusion was confirmed by visual observation of cyanosis and ST-segment elevation by continuous ECG monitoring and the reperfusion by reversal of these effects. After 40 minutes of LCA ligation, reperfusion was initiated by releasing the knot and removing the tube, and was confirmed by the appearance of epicardial hyperemic response and by ECG recovery. The chest cavity was closed and the air was expelled from the chest, sevoflurane administration was switched off and the animal was supplied with O_2_ until reflexes were detected. Analgesia was induced with meloxicam (1 mg/Kg) administered subcutaneously. Animal was placed in a cage on a heating pad until fully conscious recovery. Animals were randomly subjected to either LCA ligation or sham operation experimenting 5% mortality during the intervention. The survival rate in all groups after Ucn-2 or saline treatment was 100%. Immediately after surgery, animals were further assigned to the following experimental groups:

Group 1 (I/R; n = 6): ischemia was produced by LCA ligation during 40 minutes and the vehicle (saline 0,9% NaCl) administration by tail vein injection 5 minutes before reperfusion. Group 2 (I/R + Ucn-2; n = 5): ischemia was produced by LCA ligation during 40 minutes and an intravenous (*i.v*.) dose of Ucn-2 (50, 150, or 300 µg/Kg) was administered by tail vein injection 5 minutes before reperfusion. Group 3 (*Sham*; n = 5): operated rats underwent the same surgical procedure without coronary ligation.

Samples from I/R animal experimental models’ hearts were taken 24 hours after surgery depending on the experimental protocol.

### Cardiac function

Transthoracic echocardiographic analyses, using a Vevo^TM^ 2100 ultrasound system with transducer MS250 with a frequency range of 13-24 MHz (VisualSonics^TM^, Canada), were undertaken to detect changes in cardiac performances^55^. The basal cardiac function was analyzed 24 hours after surgery in light anesthetized rat with 2.0% sevoflurane. M-Mode images of the left ventricle at the level of the papillary muscles were obtained, and different functional hemodynamic parameters were evaluated. Images acquisition and analyses were done in a blind manner.

### Isolation of adult ventricular myocytes and simulated I/R protocol

The hearts were removed and mounted on a Langendorff perfusion apparatus. Ventricular myocytes were isolated using collagenase type II (251 IU/mL; Worthington Biochemical, Lakewood, NJ, USA) as described previously^7^. Subsequently, isolated cells were filtered, centrifuged and suspended in Tyrode solution containing (en mM): 130 NaCl, 1 CaCl_2_, 0.5 MgCl_2_, 5.4 KCl, 22 glucose, 25 HEPES, 0.4 NaH_2_PO_4_, 5 NaHCO_3_; pH was adjusted to 7.4 with NaOH. Cardiac myocytes were plated in control solution containing 1.8 mM CaCl_2_ at 37 °C, and were submitted to a protocol of I/R using a simulated ischemic solution (mM): 142 NaCl, 3.6 KCl, 1.2 MgCl_2_, 1.8 CaCl_2_, 5 NaHCO3, 20 Hepes, 20 Lactate-Na, 20 sucrose (pH 6.22). Cells were then placed during 30 minutes in an incubator at 1% O_2_ and 5% CO_2_. Afterward, cells were incubated in control solution were maintained in an incubator at 21% O_2_ and 5% CO_2_ during 18 hours. All the experiments were performed on Ca^2+^-tolerant rod-shaped myocytes at 37 °C. We considered the following experimental groups: Group 1 (Control): Untreated cardiac myocytes. Group 2 (I/R): After stabilization in control solution, cells were exposed to simulated ischemia solution during 30 minutes and reperfused for 18 hours with freshly control solution. Group 3 (Ucn-1): Same as group 2, but Ucn-1 (10 nM) was in reperfusion. Group 4, (8CPT): Same as group 3 but 8CPT (10 µM, specific agonist of Epac2) was applied instead of Ucn-1. Group 5, 6 and 7: Cells treated with either astressin (10 µM, CRF-R2 inhibitor), ESI-05 (10 µM, Epac2 inhibitor) or PD 098059 (5 µM, ERK1/2 inhibitor), 10 minutes before the addition of Ucn-1 (10 nM) following the same protocol as in group 3.

### Neonatal ventricular myocytes

Neonatal rat ventricular cardiomyocytes (NRVMs) were isolated from the hearts of 1 to 3 days-old *Wistar* rats. The heads were cut off, the auricles were discarded and the ventricular cells were dispersed by successive enzymatic digestion with 0.125% trypsin and 0.1% collagenase. NRVMs (1 × 10^6^/ml) were seeded into 6-well plates. The primary ventricular cardiomyocytes were cultured in Dulbecco’s Modified Eagle Medium DMEM/medium 199 (4:1) supplemented with 10% horse serum, 15% fetal bovine serum (FBS), 1% glutamine, 100 U/ml penicillin and 100 μg/ml streptomycin for 24 hours. On the next day, medium was replaced and cells were grown with 1% FBS. Approximately 48 hours after isolation, the cells displayed as confluent monolayer with spontaneous contractile activity. Then, the cells were cultured in medium without FBS until its use. NRVMs were subjected to the same protocol of I/R as in adult cardiac myocyte for miRNAs’ experiment.

### Western Blotting

Protein samples were extracted from cultured and treated cardiac myocytes. 40 μg of protein were subjected to SDS-PAGE (10% acrylamide) and electrotransferred onto PVDF membranes. Membranes were probed overnight at 4 °C with specific primary antibodies in TTBS with 1% of BSA. Phospho-ERK1/2 and ERK1/2 primary antibodies were from Cell Signaling (Massachusetts, USA), Epac2 antibody was from BIO*L*OG LSI (Germany). Detection was performed with the enhanced chemiluminiscence reagent ECL-plus (Amersham Bioscience, UK) in the ImageQuant LAS 4000 mini (GE Healthcare, UK). For quantification, the images were analyzed with “Image J” software using alpha-tubulin (Sigma-Aldrich, Spain) as housekeeping loading control.

### Lentivirus production and cardiac cells transfections

To ensure miRNAs mimics cells delivery, Lentivirus-expressing miR-125a-3p (LV-miR-125a-3p), Lentivirus-miR-139-3p (LV-miR-139-3p) and Lentivirus-miR-324-3p (LV-miR-324-3p) were constructed by cloning the pre-microRNA sequence in plasmid (pSIN-DUAL-GFP). Lentivirus with scramble sequence was used as control (LV-miR-control). To produce the lentiviral particles the plasmid pSIN-DUAL, together with p8.91 and pVSV plasmids were cotransfected in HEK cells using Calphos Kit (Cultek, Madrid, Spain), then purified and titled prior to their use in NRVMs cultures. The efficacy of the transfection of cultured NRVMs with lentiviral vectors conjugated with GFP was confirmed by immunofluorescence and qRT-PCR analyzes 24 hours after cells transfection (Supplement Fig. 3). Lentivirus expressing scrambled miRNAs was used as a control.

### RNA extraction

Total RNA was extracted from cultured cardiac myocytes using the RNeasy kit (Qiagen, Hilden, Germany) according to the manufacturer’s instructions. 1 µg of RNA were retro-transcribed to cDNA with the TaqMan microRNA Reverse Transcription Kit (Qiagen, Hilden, Germany). qRT-PCRs were performed using a Prism 7900HT Sequence Detection System, TaqMan primers and probes technologies (Applied Biosystems, California, USA). Fold change in gene expression was calculated using the comparative cycle threshold CT method (2^−ΔΔCT^ method) using U87 as endogenous control.

### Microarray analysis

Microarray of miRNAs was conducted using RNA samples from Langendorff perfused-hearts submitted to I/R and from heart treated with 10 nM Ucn-1 (n = 4 for each group). “Rat microRNA Microarray” from Agilent (Agilent Technologies, California, USA) was used following manufacturer’s instructions, and miRNA-expression profiling was performed using a chip containing 350 mature miRNAs. Microarray analysis was done using an Agilent’s 60-mer SurePrint scanner to acquire the microarray images, which were subsequently processed with GenePix Pro software 7.0 (Molecular Devices, California, USA) to generate the expression data in GPR (Genepix Result File) format, all according to manufacture’s instructions. The background corrected intensity values by algorithm normexp were used for analysis, and quantile normalization was employed. Expressed genes were filtered on the basis of standard deviation between four biological replicates at p < 0.05 and fold change >1.3.

To determine the biological functions and pathways regulated by miRNAs data were subjected to a bioinformatics analysis using miRBASE, PicTar, Targetscan database (http//www.mirBase.org, www.pictar.org and www.Targetscan.org) to predict target genes. Genes were selected based on: i) their appearance in the top 10 in each software; ii) their presence of at least Log values of the used software; and iii) their confirmed relation with different cardiac pathophysiological processes linked to cardioprotection, adverse remodeling, apoptosis or fibrosis among other actions.

### Statistical analysis

Data analysis was carried out using SigmaPlot software, version 11.0. A sample size calculation was performed prior the start of this study. Group data are presented as mean ± S.E.M. Single or paired Student’s t test was used to determine the statistical significance of the obtained data. The significance between multiple groups was evaluated using ANOVA followed by Tukey multiple comparison post-hoc tests. Data marked by * and ** were considered significantly different at p < 0.05, and p < 0.01 respectively.

## Electronic supplementary material

Supplementary Information
