# Supplementary material for: miR-125a, miR-139 and miR-324 contribute to Urocortin protection against myocardial ischemia-reperfusion injury
Source: Sci Rep. 2017 Aug 21;7:8898. doi: 10.1038/s41598-017-09198-x (PMC5566224; doi:10.1038/s41598-017-09198-x)
Supplement: Supplementary file 1 — Supplementary Information [file 41598_2017_9198_MOESM1_ESM.pdf]

miR-125a, miR-139 and miR-324 contribute to Urocortin protection against myocardial ischemia-reperfusion injury

Ignacio Díaz, Eva Calderón-Sánchez, Raquel Del Toro, Javier Ávila-Medina, Eva Sánchez de Rojas-de Pedro, Alejandro Domínguez-Rodríguez, Juan Antonio Rosado, Abdelkrim Hmadcha, Antonio Ordóñez, Tarik Smani.

miRNA	Predicted gene target	Full gene name	sequence ref
miR-125a-3p	Mtfr1	mitochondrial fission regulator 1	NM_001100977.1
	Igf2	insulin-like growth factor 2	NM_001190162.1
	Map3k12	mitogen activated protein kinase kinase kinase 12	NM_013055.1
	Brca1	breast cancer 1	NM_012514.1
	Xbp1	X-box binding protein 1	NM_001004210.2
	Map2k7	mitogen activated protein kinase kinase 7	NM_001025425.1
	Cpt2	carnitine palmitoyltransferase 2	NM_012930.1
	Taz	tafazzin	NM_001025748.1
	Rgs4	regulator of G-protein signaling 4	NM_017214.1
	Smad4	SMAD family member 4	NM_019275.2
	Nrg1	neuregulin 1	NM_001271130.1
miR-139-3p	Igf1r	insulin-like growth factor 1 receptor	NM_052807.2
	FoxO1	forkhead box O1	NM_001191846.2
	CxcR4	chemokine (C-X-C motif) receptor 4	NM_022205.3
	Pde4A	phosphodiesterase 4A	NM_013101.3
	Gnb1	guanine nucleotide binding protein, beta polypeptide 1	NM_030987.2
	Col5a2	collagen, type V, alpha 2	NM_053488.1
	Camta2	calmodulin binding transcription activator 2	NM_001105801.2
miR-324-3p	Crebbp	CREB binding protein	NM_133381.3
	STAT2	signal transducer and activator of transcription 2	NM_001011905.1
	Bcl2l11 (Bim)	BCL2-like 11	NM_022612.1
	Dnajb6	DnaJ (Hsp40) homolog, subfamily B, member 6	NM_001013209.1
	Nfat5	nuclear factor of activated T-cells 5	NM_001107425.1
	Dag1	dystroglycan 1 (dystrophin-associated glycoprotein 1)	NM_053697.1
	Casq1	calsequestrin 1	NM_001159594.1

Online Table 1. List of target genes for miR-125a-3p, miR-139-3p, and miR-324-3p.
List of target genes obtained by bioinformatics analysis using PicTar, TargetScan, and miRBase softwares.

miR-125a, miR-139 and miR-324 contribute to Urocortin protection against myocardial ischemia-reperfusion injury

Ignacio Díaz, Eva Calderón-Sánchez, Raquel Del Toro, Javier Ávila-Medina, Eva Sánchez de Rojas-de Pedro, Alejandro Domínguez-Rodríguez, Juan Antonio Rosado, Abdelkrim Hmadcha, Antonio Ordóñez, Tarik Smani.

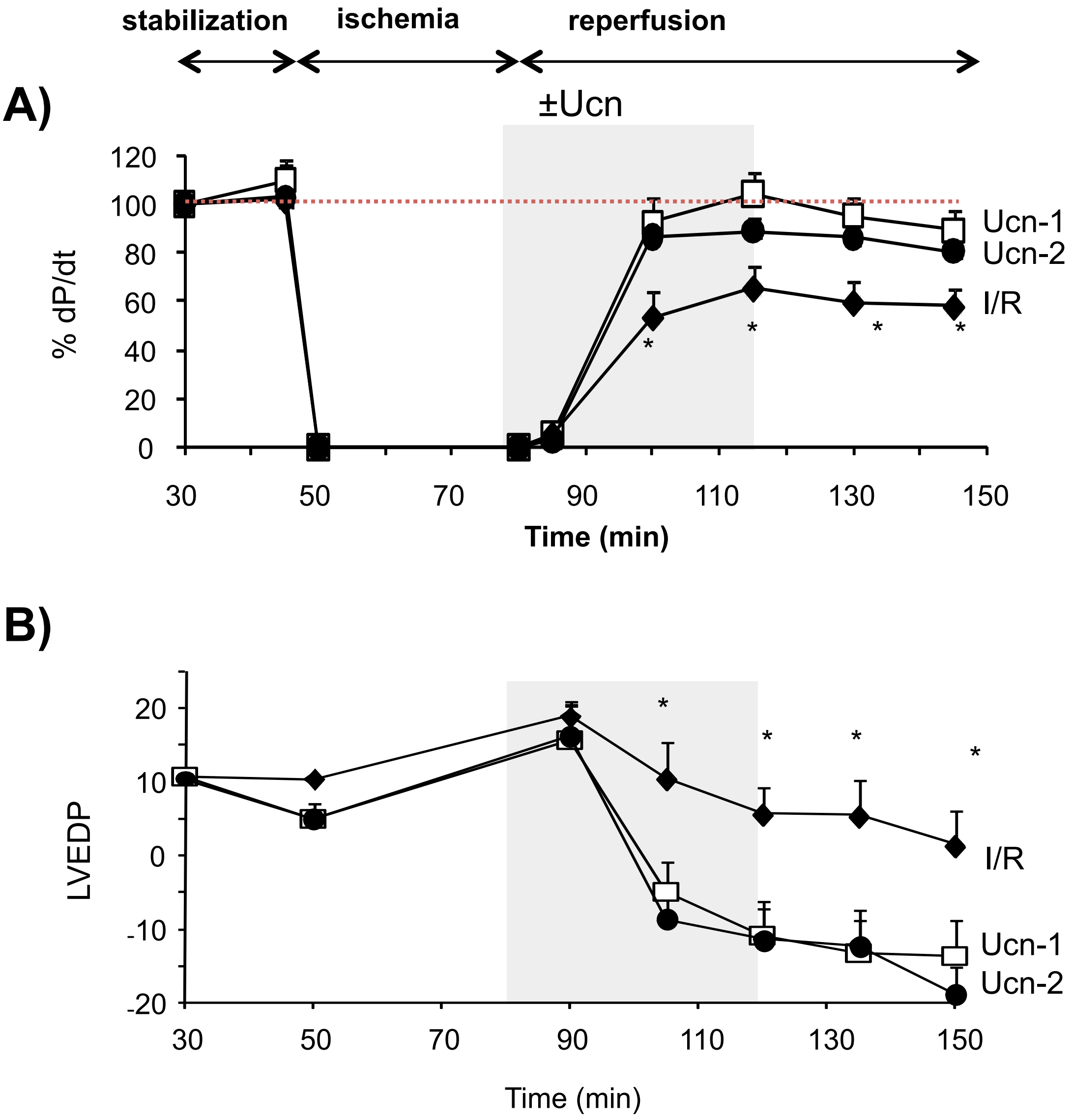

Online Figure 1. Comparative effects of Urocortin-1 and Urocortin-2 in Langendorff-perfused rats hearts

A) Graph shows summary data of the contractility expressed as +dP/dt (%) in hearts subjected to ischemia/reperfusion protocol (I/R, full diamond, n = 6), in hearts treated with 10 nM Ucn-1 (open square, n = 6); and in heart treated with Ucn-2 (full circle, n = 6). Ucn-1 or Ucn-2 were applied 3 minutes before ischemia ends, and it continuous during the first 30 minutes in reperfusion as indicated by area in grey. B) Shows summary data of the left ventricular end diastolic pressure (LVEDP, mmHg) in the same conditions as in "A". "*" indicates significance at $p<0.05$ of I/R vs Ucn-1 or Ucn-2.

miR-125a, miR-139 and miR-324 contribute to Urocortin protection against myocardial ischemia-reperfusion injury

Ignacio Díaz, Eva Calderón-Sánchez, Raquel Del Toro, Javier Ávila-Medina, Eva Sánchez de Rojas-de Pedro, Alejandro Domínguez-Rodríguez, Juan Antonio Rosado, Abdelkrim Hmadcha, Antonio Ordóñez, Tarik Smani.

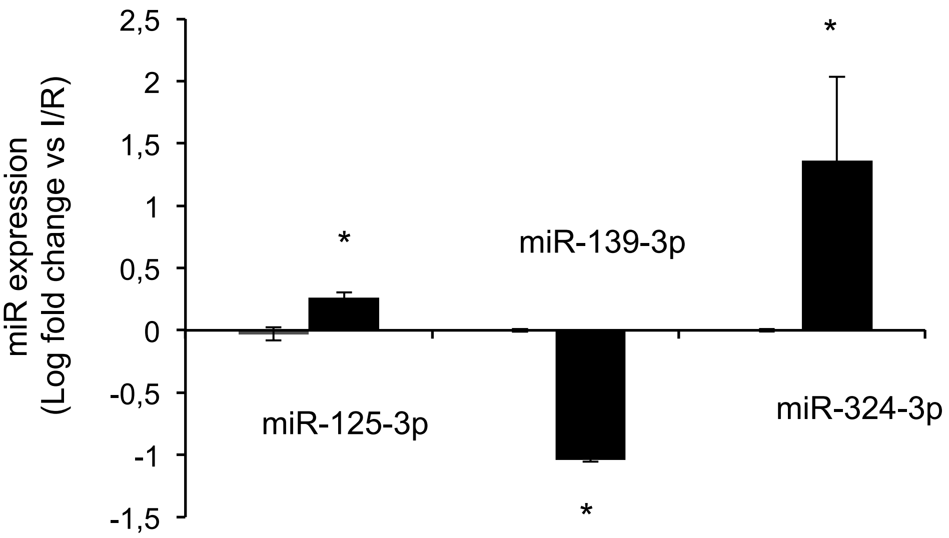

Online Figure 2. Urocortin-1 regulates the expression of miR-125-3p, miR-139-3p and miR-324-3p in neonatal ventricular cardiac myocyte.
Graph shows the expression of miR-125, miR-139 and miR-324 in cardiac myocyte undergoing the protocol of I/R and treated with Ucn-1 (10 nM) in reperfusion. Values are shown in logarithmic scale and are means ± S.E.M, (n = 3 independent cells preparation). "*" indicates significance at $p<0.05$.

miR-125a, miR-139 and miR-324 contribute to Urocortin protection against myocardial ischemia-reperfusion injury

Ignacio Díaz, Eva Calderón-Sánchez, Raquel Del Toro, Javier Ávila-Medina, Eva Sánchez de Rojas-de Pedro, Alejandro Domínguez-Rodríguez, Juan Antonio Rosado, Abdelkrim Hmadcha, Antonio Ordóñez, Tarik Smani.

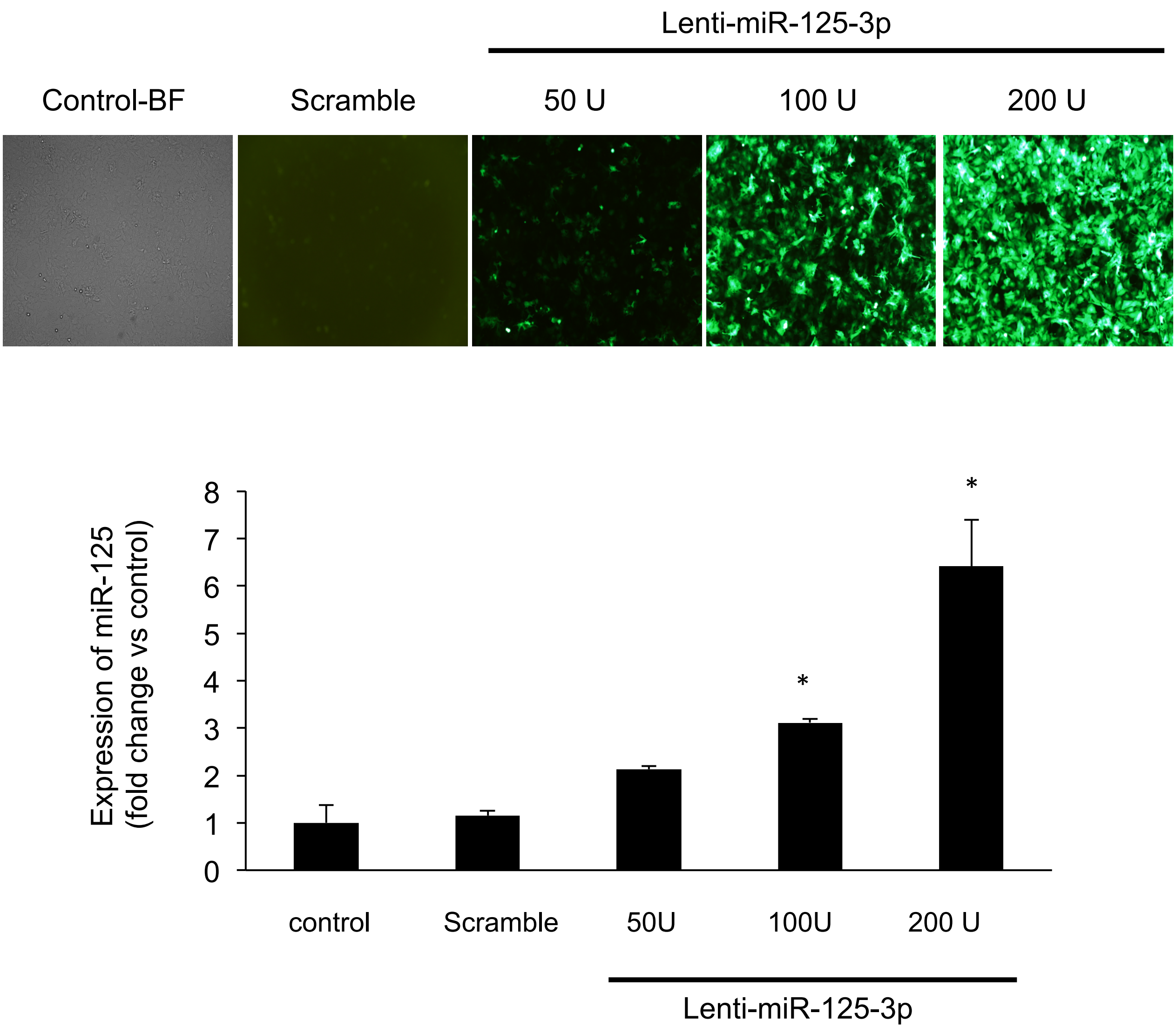

Online Figure 3. Experiments showing the efficiency of cardiac cell transfection with lentivirus.

A) Images of rat neonatal cardiomyocyte in the following condtions: control non transfected cells; cells transfected with lentivirus expressing scrmable miRNA; cells transfected with increasing doses of lentivirus overexpressing miR-125-3p.
B) Graph showing the expression of miR-125-3p noemalized to control value examined by q-RT-PCR. . Values aree means ± S.E.M, (n =). “*” indicates significance at $p<0.05$ vs control.
